# Supplementary material for: Human Non-Small Cell Lung Cancer-Chicken Embryo Chorioallantoic Membrane Tumor Models for Experimental Cancer Treatments
Source: Int J Mol Sci. 2023 Oct 21;24(20):15425. doi: 10.3390/ijms242015425 (PMC10607033; doi:10.3390/ijms242015425)
Supplement: Supplementary file 1 [file ijms-24-15425-s001.zip › ijms-2638714-supplementary.pdf]

Supplementary data File

Human Non-small Cell Lung Cancer - Chicken Embryo Chorioallantoic Membrane Tumor Models For Experimental Cancer Treatments

Jing Li, Tereza Brachtlova, Ida H. van der Meulen-Muileman, Stijn Kleerebezem, Chang Liu, Peiyu Li and Victor W. van Beusechem

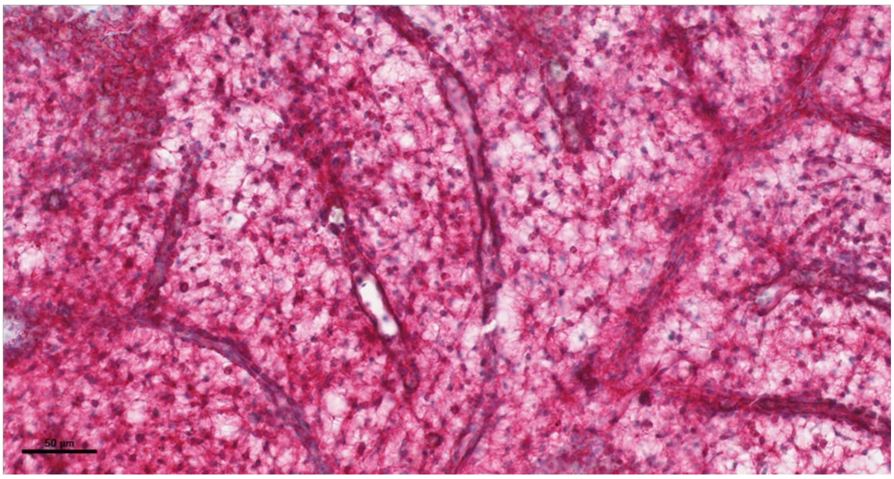

Figure S1. LCA staining of CAM tissue from a fertilized chicken egg without transplanted NSCLC cells. The section was counterstained with hematoxylin. Most intense LCA staining (red) is observed in endothelial cells. Scale bar is 50 μm.

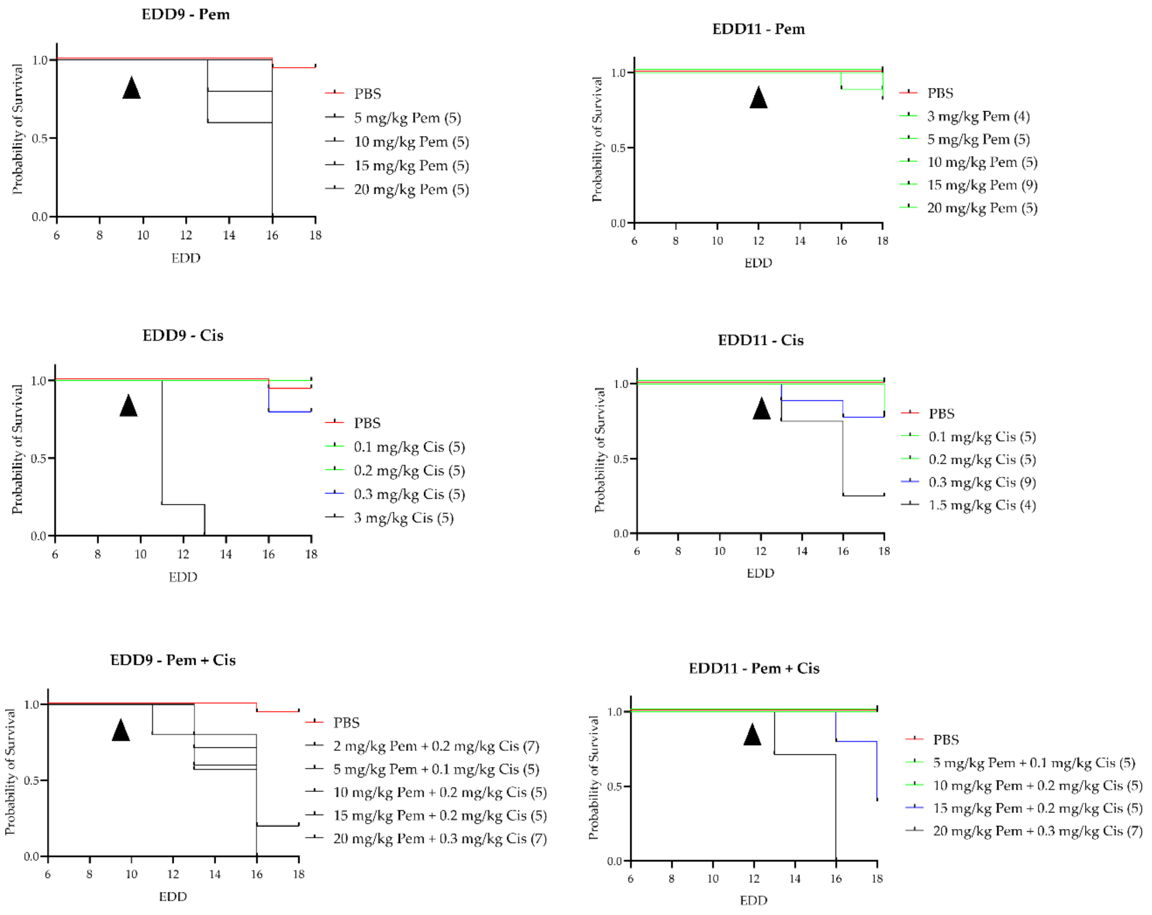

Figure S2. Embryo lethality upon systemic chemotherapy treatment. Pemetrexed (Pem) and/or Cisplatin (Cis) were administered at the indicated dose (in mg/kg) onto the CAM of viable fertilized chicken eggs on EDD 9 (left panels) or EDD 11 (right panels). Embryo viability was assessed until EDD 18 and survival probabilities were documented. Number of eggs included in each treatment group are given between brackets. At both time points, 17 PBS controls were included and shown for comparison in each panel. Arrowheads, days of chemotherapy treatment. Red lines, PBS controls. Green lines, non-lethal chemotherapy treatments (survival not substantially deviating from PBS controls). Blue lines, treatments with medium lethality. Black lines, highly lethal treatments (i.e., majority of embryos not surviving until EDD 18).

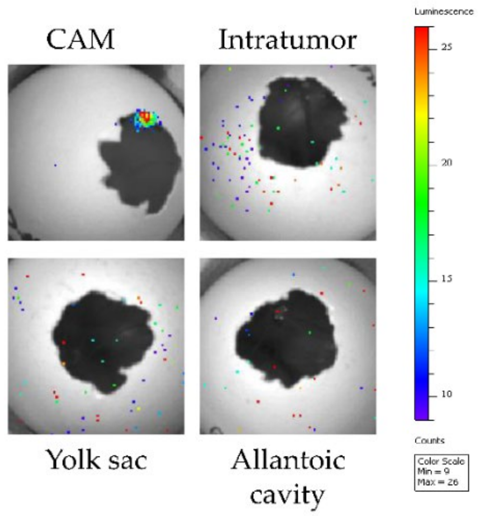

Figure S3. Comparison of different lentiviral vector delivery routes to NSCLC-CAM tumors. LV-FLuc (1x10^6 TU for the intratumoral injection route or 1x10^7 TU for the other 3 routes) was administered to eggs bearing SW1573 tumors on EDD14 and luminescence was measured on EDD16. Examples of 4 eggs analyzed per group are shown. Luminescence at the location of the tumor was detected only upon delivery to the CAM, albeit in a minority of the eggs.

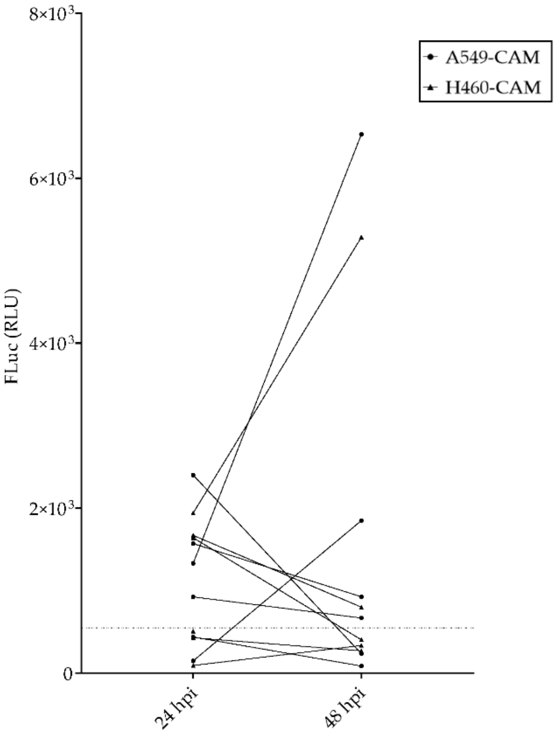

Figure S4. Quantification of luminescence emitted from A549 or H460-CAM tumors transduced by a single administration of 1x10^9 copies LV-Fluc to the CAM and measuring luminescence at 24 hpi and 48 hpi. Graph shows pairwise comparisons of luminescence measurements on 10 tumors at the two time points. ROI: 30 pixels diameter. The dashed line indicates the maximum background measured in these experiments (550 RLU). Mean luminescence signals do not change between 24 hpi and 48 hpi (NS, paired t-test).

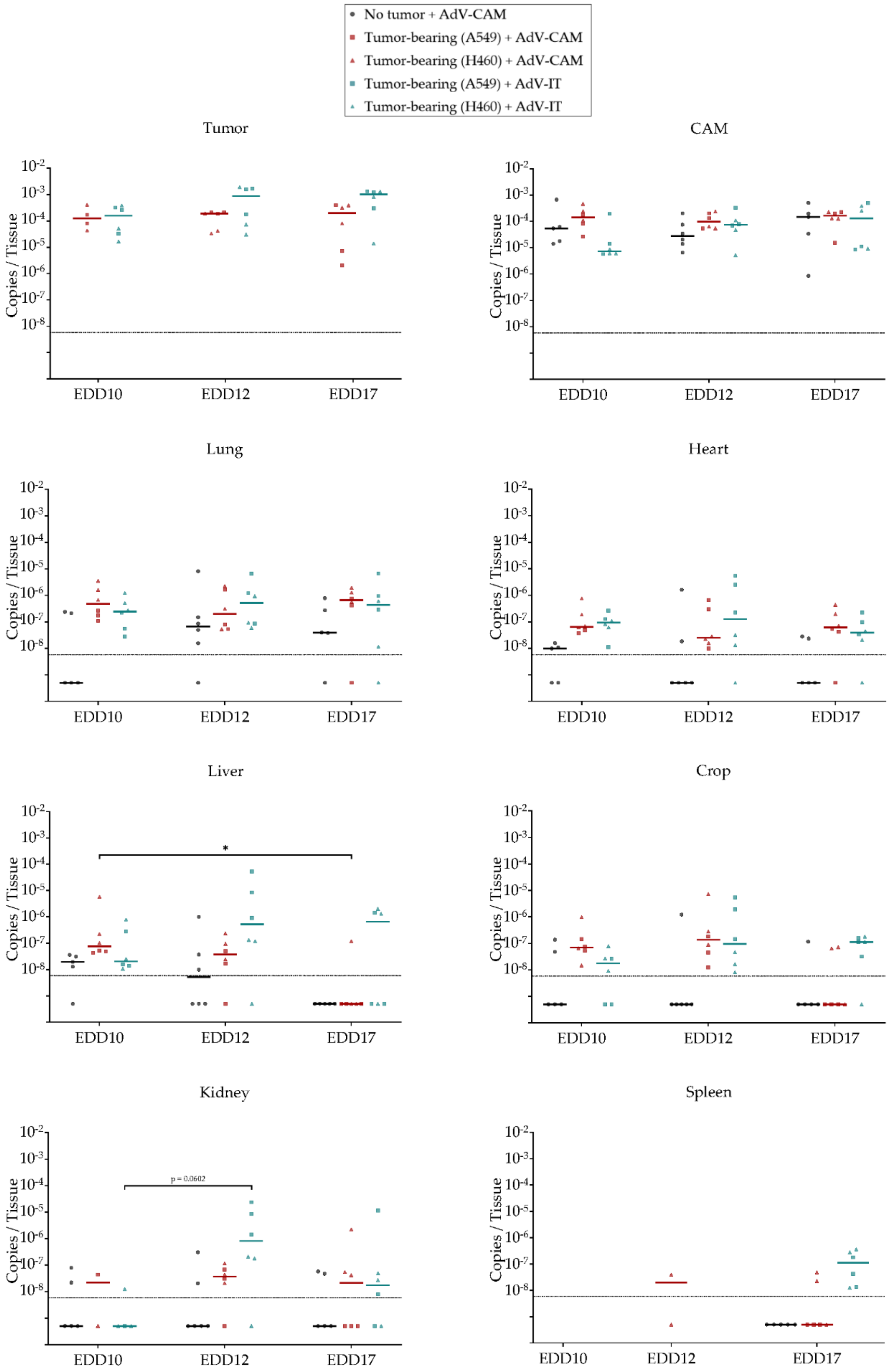

Figure S5. Biodistribution of AdCMV-Luc administered at 3 different time points to chicken embryos. Same data as shown in Figure 8, reorganized in separate panels per analyzed tissue. Significant differences between the same treatments on different EDDs as tested using Kruskal-Wallis test are shown. *, $p<0.05$.

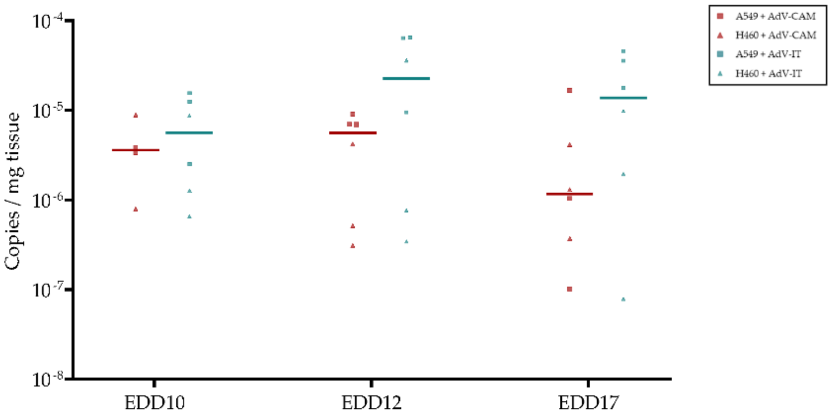

Figure S6. Comparison of tumor tissue transduction efficiencies after AdCMV-Luc administration on different EDDs. To compensate for tumor growth, luminescence intensities are given per mg tissue. Differences were not significant (Kruskal-Wallis test).

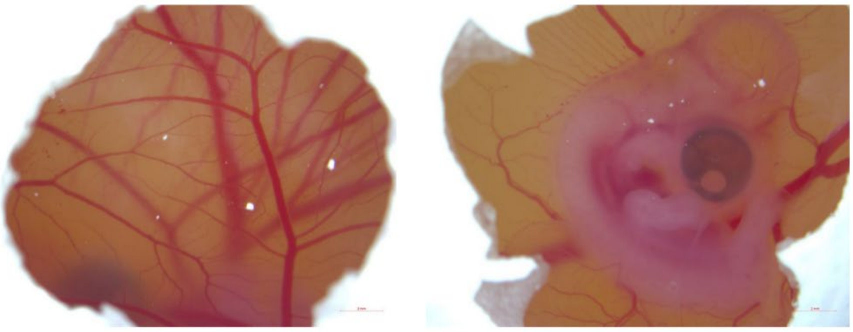

(a) (b)

Figure S7. Microscopic images of differently developed vessel networks on EDD6. (**a**) A well-developed vessel network. (**b**) A poorly developed vessel network. Images were taken through the shell window at 6.3x magnification. Only eggs with well-developed vessel networks were used to grow NSCLC tumors.

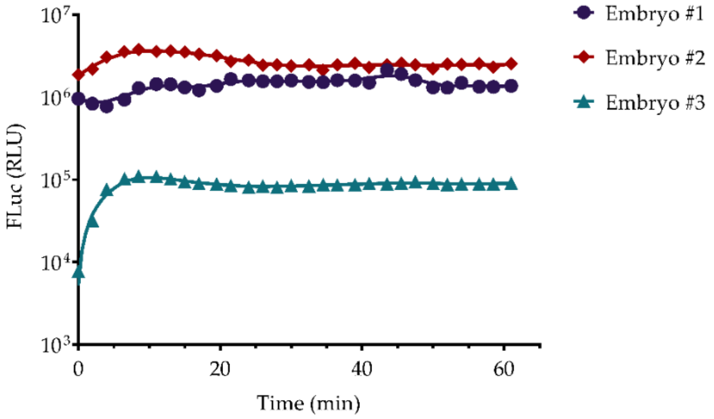

Figure S8. Luminescence kinetics after D-luciferin substrate application onto the CAM. Firefly luciferase-expressing reporter cell line SW1573-Fluc was made by transducing SW1573 cells with LV-FLuc in the presence of 4 µg/ml polybrene and selecting cells resistant to 0.5 µg/ml puromycin. SW1573-Fluc CAM tumors were established using the standard procedure for NSCLC-CAM tumors. On EDD11 (embryo#1), 14 (embryo#2), or 15 (embryo#3), 30 microliter 30 mg/ml D-luciferin was applied onto the CAM and luminescence was imaged every 2 minutes for more than one hour. Stable luminescence signals were observed after 20 minutes, remaining constant for at least another 40 minutes. Similar kinetics were observed for tumors with various luminescence levels.

Table S1. Mutations in NSCLC-associated genes in the five NSCLC cell lines used to grow NSCLC-CAM tumors.

Cell line		A549	H1299	H292	H460	SW1573
NSCLC Characterization		Adenocarcinoma	Large Cell Carcinoma	Mucoepidermoid Carcinoma	Large Cell Carcinoma	Alveolar Cell Carcinoma
COSMIC Sample ID		COSS905949	COSS724831	COSS753604	COSS905943	COSS724878
Number of Cancer Gene Census entries		44	52	53	46	66
Cancer Gene Census genes and gene families with known mutations in NSCLC	KRAS	p.G12S, c.34G>A (homozygous)			p.Q61H, c.183A>T (homozygous)	p.G12C, c.34G>T (homozygous)
	NRAS		p.Q61K, c.181C>A (heterozygous)			
	ERBB	ERBB3: c.1480+42G>T (heterozygous) ERBB3: c.1859+23G>T (heterozygous)			ERBB4: c.2867-107G>T (heterozygous) ERBB4: c.2867-111T>G (heterozygous) ERBB4: c.1124+95A>T (heterozygous)	
	NRG1		p.N383S, c.1148A>G (heterozygous)			
	NF1		c.4836-7170del (heterozygous) c.4836-6997_4836-6990del (heterozygous)		c.4836-5376T>G (heterozygous)	
	STK11	p.Q37*, c.109C>T (homozygous)			p.Q37*, c.109C>T (homozygous)	
	TP53		p.0, c.1_*del (whole gene deletion)			
	CDKN2A				c.*151-104T>C (homozygous)	
	PIK3CA				p.E545K, c.1633G>A (heterozygous)	p.K111E, c.331A>G (heterozygous)
	ABL		ABL1: c.1142+40G>C (heterozygous) ABL1: c.1045+101G>T (heterozygous) ABL2: c.157+45311G>C (heterozygous)			
	RB1			c.264+11031C>G (heterozygous) c.265-14129C>T (heterozygous)		
	NTRK3					c.765+89A>G (heterozygous)
	MAP2K1 (MEK1)				p.Y134C, c.401A>G (heterozygous)	p.Q56P, c.167A>C (heterozygous)
	SMAD4			c.667+54T>C (heterozygous)		
	LTK		p.P463R, c.1388C>G (homozygous)			

The list includes mutations in Cancer Gene Census genes that are reported in at least one of the cell lines. NSCLC-associated genes in which no mutation was found in any of the cell lines include EGFR, ERBB2/Her-2, ALK, BRAF, HRAS, MET, RET, ROS1. Data were extracted from the Catalogue Of Somatic Mutations In Cancer (COSMIC) Cell Lines Project v98 (released 23 May 2023). For each cell line, the COSMIC Sample ID is given as well as the current number of Cancer Gene Census entries. Complete and up to date mutational profiles of the NSCLC cell lines are available from www.sanger.ac.uk/tool/cosmic/.
